# A 3D Camera-Based Approach for Real-Time Hand Configuration Recognition in Italian Sign Language

**DOI:** 10.3390/s26031059

**Published:** 2026-02-06

**Authors:** Luca Ulrich, Asia De Luca, Riccardo Miraglia, Emma Mulassano, Simone Quattrocchio, Giorgia Marullo, Chiara Innocente, Federico Salerno, Enrico Vezzetti

**Affiliations:** 1 Management and Production Engineering, Politecnico di Torino, C.so Duca degli Abruzzi, 24, 10129 Torino, Italy; 2 Biomedical Engineering, Politecnico di Torino, C.so Duca degli Abruzzi, 24, 10129 Torino, Italy

**Keywords:** sign-language recognition, Italian Sign Language (LIS), machine learning, RGB-D cameras, MediaPipe

## Abstract

Deafness poses significant challenges to effective communication, particularly in contexts where access to sign language interpreters is limited. Hand configuration recognition represents a fundamental component of sign language understanding, as configurations constitute a core cheremic element in many sign languages, including Italian Sign Language (LIS). In this work, we address configuration-level recognition as an independent classification task and propose a machine vision framework based on RGB-D sensing. The proposed approach combines MediaPipe-based hand landmark extraction with normalized three-dimensional geometric features and a Support Vector Machine classifier. The first contribution of this study is the formulation of LIS hand configuration recognition as a standalone, configuration-level problem, decoupled from temporal gesture modeling. The second contribution is the integration of sensor-acquired RGB-D depth measurements into the landmark-based feature representation, enabling a direct comparison with estimated depth obtained from monocular data. The third contribution consists of a systematic experimental evaluation on two LIS configuration sets (6 and 16 classes), demonstrating that the use of real depth significantly improves classification performance and class separability, particularly for geometrically similar configurations. The results highlight the critical role of depth quality in configuration-level recognition and provide insights into the design of robust vision-based systems for LIS analysis.

## 1. Introduction

Deafness is a degree of hearing loss that prevents a person from understanding speech, even with amplification; however, it should not be considered solely as a medical condition. Deafness also has significant social implications, as it affects not only communication but also interpersonal relationships and social inclusion [1]. The existing literature highlights persistent challenges in establishing effective communication with deaf individuals, particularly in healthcare contexts. One major issue is the limited access to health-related information within the deaf community [2], which contributes to their under-representation in healthcare service research [3]. For example, older adults with hearing loss are more likely to be hospitalized than their hearing peers, often due to inadequate understanding of their specific needs [4]. Moreover, many deaf individuals have lower reading proficiency and may rely on non-standard written language. For these reasons, the provision of sign language interpretation services is often essential to prevent misunderstandings and ensure equitable access to care [5].

Sign language is a form of communication that relies on visual signals rather than acoustic sound patterns. Approximately 60 sign languages are currently recognized and used worldwide [6]. Among them, some languages remain underrepresented in research, including Italian Sign Language (LIS). The literature shows that most studies focus on American Sign Language (ASL) and British Sign Language (BSL), while comparatively few contributions address LIS [7]. A deeper understanding of LIS structure is therefore essential to support the development of effective translation and recognition systems, particularly for applications targeting the Italian deaf community [8,9]. In addition, individuals who have learned LIS often exhibit lower social anxiety and higher levels of life satisfaction and self-esteem compared to those who have not [1].

From an articulatory perspective, signs may consist of manual components, bodily components, or a combination of both. The manual component, which may involve one or two hands, is characterized by four fundamental elements, known as cheremes: (1) hand configuration, referring to the shape assumed by the hand during articulation; (2) palm orientation; (3) location, describing the position of the hands relative to the signer’s body; and (4) hand movement. Meaning in sign language emerges from combinations of these components, and changes in any one of them can alter the sign’s interpretation [10]. Among the possible hand configurations, a subset known as unmarked configurations plays a particularly important role. In LIS, this group includes six configurations: A, B, 5, O, C, and G. These configurations are considered fundamental because they are biomechanically simple to produce and are therefore widely used by the signing population. Their simplicity and visual distinctiveness also make them the first configurations learned by children during the acquisition of LIS. A more detailed discussion of these configurations is provided in Section 3.3.

A wide range of technologies has been developed for sign language recognition, with most approaches relying on artificial intelligence (AI) and, more specifically, machine learning (ML) techniques. Methods such as Support Vector Machines (SVMs) [11,12], Convolutional Neural Networks (CNNs) [12,13], and Long Short-Term Memory (LSTM) networks [14] have been successfully applied in different application domains, including healthcare, to facilitate communication with deaf individuals. These approaches rely on various acquisition modalities, ranging from sensor-based systems, such as instrumented gloves [15], to vision-based systems using RGB or RGB-D cameras, including devices such as the Kinect V2 [13,16]. Additional methods focus on handcrafted feature extraction, including Common Spatial Patterns (CSPs) [17] and Histogram of Oriented Gradients (HOG) [18].

Hand gesture recognition systems may target either dynamic gestures [19,20,21] or static gestures [22,23]. The current state of the art is highly heterogeneous, as recognition methods are often tailored to the linguistic, cultural, and environmental characteristics of a specific sign language. Consequently, no single gesture-recognition approach can be considered universally applicable. Instead, effective systems must account for language-specific properties and usage contexts [24].

Most existing systems focus on recognizing individual signs or fingerspelled alphabet letters, which may be insufficient for capturing the structure of natural communication. Recent studies have therefore emphasized the importance of recognizing hand configurations as an intermediate linguistic unit, enabling a more comprehensive understanding of signed discourse [25]. Although recognizing individual letters has been widely explored [22,26,27], this approach often results in slow and cognitively demanding communication.

In this context, the present work addresses the problem of recognizing static hand configurations in Italian Sign Language, with the goal of supporting real-time, vision-based communication systems tailored to LIS. The specific objectives of the study are:

1. To formulate hand configuration recognition as an independent, configuration-level classification problem, decoupled from temporal gesture modeling;
2. To design a vision-based recognition framework that integrates MediaPipe hand landmarks with three-dimensional geometric features derived from depth data;
3. To assess the impact of sensor-acquired RGB-D depth compared to estimated depth on classification accuracy and class separability.

While several previous studies address sign language recognition by decomposing signs into multiple phonological or sub-lexical components, the objective and formulation of the present work differ in a fundamental way. Phonological decomposition approaches typically aim to jointly model hand configuration, movement, orientation, and location as interconnected elements contributing to the recognition of complete signs or sign sequences. In contrast, this study deliberately isolates hand configuration as an independent classification target, treating it as a standalone cheremic unit rather than as one attribute within a broader phonological representation. The proposed framework does not attempt to infer sign identity or reconstruct full phonological structures; instead, it focuses on learning a direct mapping between three-dimensional hand landmark geometry and discrete configuration classes. This configuration-level formulation enables the development of modular recognition components that can be reused, combined, or integrated into higher-level sign language understanding systems, while also allowing a controlled analysis of the impact of depth information on geometric discriminability without confounding effects from other linguistic dimensions.

The rest of this paper is organized as follows: Section 2 provides a detailed state of the art, reviewing existing technologies and their applications in sign language recognition. Section 3 describes the methodology used in developing the proposed system, including the ML algorithms and data processing. Section 4 presents the results, highlighting the system’s effectiveness in sign recognition and limitations of this work. Finally, Section 5 concludes with a summary of key results and suggestions for future research.

## 2. Related Work

Technology has always been a crucial tool for enhancing communication quality and in the last decade, numerous systems for the automatic recognition of sign language have been proposed. This rapid evolution is driven by the growing emphasis on creating an inclusive society and the increasing significance of hand gesture recognition in human–robot interaction for improving industrial processes [16,23].

Methods for extracting hand features can be divided into intrusive and non-intrusive categories. The former relies on physical sensors posed on the signer to achieve the correct individuation of hand landmarks. The latter accomplishes this task only through images, without expensive instrumentation. For this purpose, in [28], the authors presented a sensored glove system to perform sign language recognition based on hand kinematics assessment. A radial basis function kernel SVM was used for recognition, achieving an average recognition rate of 96.7%.

Other approaches rely on computer-vision methods and several studies have been conducted in this field over the past decade. While intrusive systems require interference with the signer, such as using colored or electronic gloves, non-intrusive systems use vision-based recognition approaches. These approaches do not require wearable sensors. Instead, features are extracted from RGB and depth images for classification, enabling seamless communication and easy acquisition. Useful features are extracted from the collected data to distinguish various hand gestures. For example, in [22], an ASL alphabet letters recognition system was developed by processing input data from RGB cameras and using the least Euclidean distance between a feature vector created with a feature extraction algorithm and training data of feature vectors. Camera-based methods have benefited from the increased use of several algorithms for classification based on AI, which can be divided into ML and Deep Learning (DL) approaches.

Regarding ML techniques, in [29] a methodology for feature extraction and decomposition of Brazilian Sign Language (BSL) signs into their phonological structure is presented. This method involves an RGB-D sensor to obtain intensity, position, and depth data. Then, SVM is used to classify signs based on these features and linguistic elements. The experiments show that the attributes of these elements can be successfully recognized in terms of the features obtained from the RGB-D images, with accuracy results individually above 80% on average. In this work, the authors address sign language recognition by exploiting phonological structure, jointly modeling hand configuration together with movement, orientation, and location using RGB-D data. In contrast, the present work isolates hand configuration as an independent classification target, decoupling it from other phonological components. This configuration-level formulation does not aim at sign recognition or phonological reconstruction, but rather at learning a modular mapping between three-dimensional hand landmark geometry and discrete LIS configuration classes, enabling a focused analysis of depth information on geometric discriminability.

In [30], the work was based on the open-source MediaPipe framework and studied which ML algorithms were better at simplifying Sign Language Recognition (SLR). Comparing different ML algorithms, it is clear that the SVM algorithm outperforms other ML techniques in effectiveness and accuracy, and it is extensively used in the recent literature. In [31], a 3D dynamic gesture recognition approach explicitly targeted at leap motion data is proposed. Spatial feature descriptors based on the positions of fingertips and the palm center are extracted and fed into an SVM classifier, achieving an accuracy rate of about 81%. Similarly, in [20], a Vietnamese Sign Language (VSL) recognition system was proposed. The Microsoft Kinect depth sensor captured input data. The feature extraction stage was performed by dividing the volume into a 3D grid of same-size blocks, each converted into a scalar value. SVM was employed, and the Hidden Markov Model (HMM) technique was also applied for comparison. A dataset of 30 dynamic gestures in VSL was created by five volunteers, yielding promising results with an average accuracy of up to 95%. In [32], M. Tun et al. proposed a real-time sign language recognition system to translate 30 static sign gestures into Myanmar alphabets. Principal Component Analysis (PCA) performed feature extraction and an SVM recognized Myanmar Sign Language (MSL) gestures, resulting in successful recognition accuracy of static sign gestures of MSL alphabets at 89%. Finally, in [23] the authors proposed a gesture recognition algorithm for robotic systems based on the Histogram of Oriented Gradient (HOG) incorporating SVM, showing improved accuracy of the proposed algorithm up to 99%.

In recent years, the rapid development of DL has led to its increasing use in gesture and sign language recognition research. Deep features tend to have better spatial–temporal representation and can model more sign language actions and movement changes. Neural network models, including CNNs, Recurrent Neural Networks (RNNs), and Graph Neural Networks (GNNs), have all been used in sign language research [33]. In [34], a sentence-level sign language recognition method using DL was proposed, combining a 3D CNN to extract features from each video frame and a Bidirectional RNN (Bi-RNN) to extract the sequential features of the video frames, ultimately generating a possible sentence. In [13], an implementation of Self Multi-Layer Perceptron (MLP)-infused CNN-LSTM models on a multi-modality video dataset for Arabic Sign Language recognition was proposed, achieving 86.97% average accuracy and 87.69% overall accuracy. In [19], a 3D dynamic hand gesture dataset providing sequences of hand skeletal data and depth images was collected. Two recognition methods were used: Skeleton-based Dynamic hand gesture recognition and CNN, demonstrating promising results with accuracy rates of 88.24% and 81.90%, for 14 and 28 different gestures, respectively.

Italian Sign Language (LIS), American Sign Language (ASL), and Brazilian Sign Language (BSL) are distinct natural languages with independent historical origins and linguistic structures. In addition to methodological differences, it should also be noted that ASL and BSL are not mutually intelligible, and LIS does not derive from either of them, exhibiting its own lexicon, morphological rules, and syntactic organization. While all three languages share the use of common manual parameters such as hand configuration, orientation, location, and movement, the inventory and functional role of specific hand configurations differ across languages. In particular, certain configurations considered unmarked or frequent in LIS may have different grammatical functions or usage frequencies in ASL and BSL [35]. Despite this, the majority of vision-based sign recognition studies have focused on ASL and BSL, with comparatively limited attention to LIS-specific configuration modeling. These linguistic differences imply that sign recognition systems developed for ASL or BSL cannot be directly transferred to LIS without adaptation, motivating the need for language-specific configuration-level analysis.

## 3. Materials and Methods

**Figure 1:**
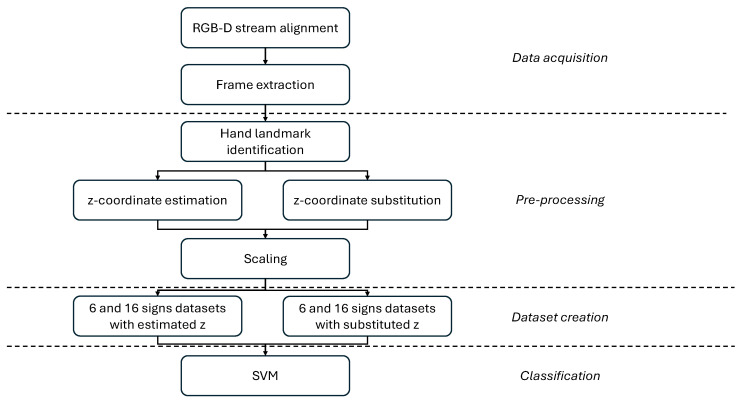
Workflow of gesture recognition.

The current investigation aimed to correctly classify single unmarked hand configurations from RGB-depth data. The workflow is reported in Figure 1 and described in the following Sections.

### 3.1. Data Acquisition

Both color images (RGB) and depth information were acquired through a low-cost RGB-D camera. These cameras offer significant advantages by providing both color (RGB) and depth (D) information in a single device, enabling a richer understanding of the scene compared to standard RGB cameras. This combined data allows for accurate 3D perception, making it possible to detect object shapes, positions, and distances, which is essential for applications involving human–computer interaction and gesture recognition. Additionally, their affordability makes advanced 3D sensing accessible, lowering the barrier to experimenting with 3D reconstruction, motion tracking, and object detection.

The depth data were acquired using an Intel RealSense Depth camera SR305 (Intel Corporation, Santa Clara, CA, USA) [36]. This device is a coded-light 3D camera that provides a depth resolution of up to 640 × 480 pixels at 30 frames per second (FPS), with an operating range typically between 0.2 m and 1.5 m. Depth and RGB cameras were set with the same video specifics to achieve the alignment between the two streams. The recording setup featured a computer Acer Nitro AN515-58 (Acer Inc., New Taipei City, Taiwan) equipped with Intel RealSense Viewer (version 2.53.1), enabling real-time data processing of RGB and depth data streams. The real-time system achieves 28–30 FPS on HoloLens 2 (Snapdragon 850, 8-core HPU) with 33 ms user-perceived latency, suitable for live guidance. Windows Device Portal shows higher and variable latency due to streaming, not native performance. Nonetheless, this affects only an eternal observer, not the user performing the application.

RGB and depth frames were extracted offline from the experimental recordings acquired during the data collection sessions. This step was performed prior to the training and evaluation phases. Offline extraction was adopted to eliminate real-time processing constraints and potential computational limitations, ensuring consistent data quality across all samples.

**Figure 2:**
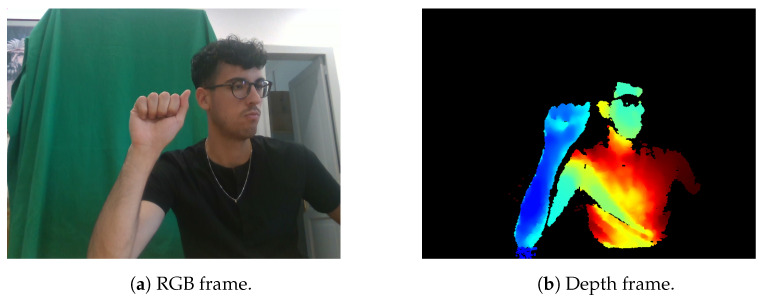
Examples of acquired frames.

Two researchers manually selected the RGB (Figure 2a) and depth frames (Figure 2b). Only visually clear and semantically representative frames were retained for the final dataset.

### 3.2. Pre-Processing

The pre-processing phase involved the identification of hand landmarks, the z-coordinate substitution, and a scaling process.

**Figure 3:**
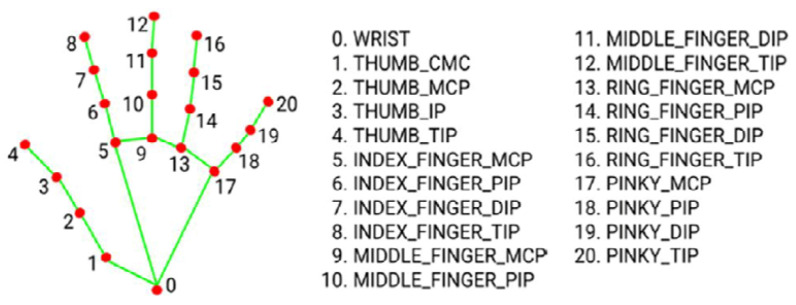
Hand landmarks extracted from Mediapipe.

Mediapipe Hand library (version 0.10.32) was exploited to extract the hand landmarks. Mediapipe [37] is Google’s open-source framework designed primarily for ML pipeline implementation. It uses a pre-trained neural network architecture for hand-landmark recognition from RGB images. The landmark coordinates detected on the hand are 21 points, each registered with *x*, *y*, and *z* values, corresponding to the width, height, and depth of the location of the corresponding landmark. A comprehensive chart is shown in Figure 3.

*x* and *y* coordinates range from 0 to 1 because they are normalized according to the image dimensions. The *z* value is an estimated value, typically not sufficiently accurate [38]. The MediaPipe landmarks were organized into a data frame and used as SVM input. The *pandas* library (version 2.2.3) in Python (version 3.9.13), widely utilized for data manipulation, was employed to construct this data frame. This choice was made due to this library’s capability to create a two-dimensional labeled data structure. Additionally, its flexibility allowed for including a column containing the configuration of the analyzed frame, thereby creating a supervised dataset. To meet the requirements of the SVM, categorical labels were transformed into numerical values suitable for ML algorithms. Finally, the data were compiled into a matrix of size nx64 and stored in a *.csv* format file, where *n* represents the number of extracted frames, and 64 corresponds to the triplets of coordinates (*x*, *y*, *z*) of the 21 landmarks plus the label name. An example of this data structure is illustrated in Table 1.

**Figure 4:**
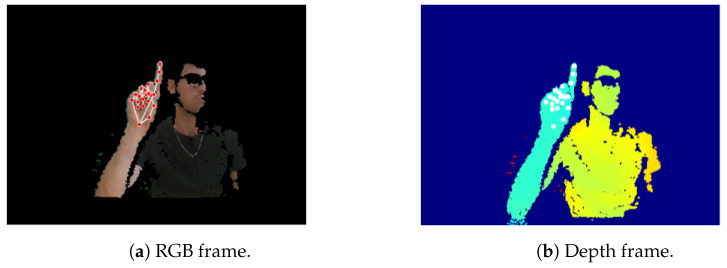
Correspondence among aligned RGB and depth frames.

To improve the depth information [39], the estimated MediaPipe z values have been replaced by the corresponding depth frame data obtained from the camera. Due to the misalignment of RGB and depth sensors in the RGB-D camera setup, alignment of depth and RGB frames was essential. By utilizing information about the relative positions of the two cameras, each landmark’s *x* and *y* coordinates were paired with their respective depth values. After successfully performing the alignment, the MediaPipe algorithm was applied to the RGB image to obtain the coordinates for each landmark, as shown in Figure 4a. Subsequently, the estimated z-coordinate values were replaced with the corresponding depth values from the depth map (Figure 4b). Depth values extracted from the RGB-D camera were converted into metric units using the scale factor provided by the device specifications. This conversion ensures physical consistency across frames and sessions while supporting reproducibility across different sensing configurations. The use of meters aims to maintain correct relative depth relationships among landmarks. For the sake of clarity, since all features are subsequently standardized to zero mean and unit variance prior to classification, the absolute scale of the depth values does not influence the learning process or classification performance. Consequently, the conversion to metric units serves as a normalization and interoperability choice rather than a modeling assumption related to hand dimensions.

Ensuring the correct substitution of z-values was a key step. Therefore, a control mechanism was introduced to check each frame for landmarks with z-values equal to 0, which indicates incorrect substitution due to alignment errors or missing depth values from the depth camera. For such landmarks, the neighboring pixels for corresponding z-values were checked. If a neighboring z-value was available, it was used as a substitute, accounting for potential approximation errors. If more than three landmarks in a frame had unsubstituted z-values, the frame was removed from the newly created database to avoid confusing data for the classifier.

In Section 4, results will be provided for both data using the estimated depth and data with depth values substituted with those ones directly acquired from the camera. This choice was motivated by the fact that the literature reports conflicting evidence regarding the reliability of depth information obtained from low-cost 3D cameras [8]. For our work, accurate depth information was crucial because the gestures were recorded not only with the hand perfectly aligned to the camera. In fact, although the experiment was conducted under controlled conditions, it was specifically designed to evaluate the performance of our methodology in non-ideal scenarios, reflecting more realistic usage conditions.

The coordinates standardization was performed by removing the mean and scaling to unit variance, ensuring that the model learns the gesture patterns rather than the absolute positions. To evaluate the effectiveness of this operation, the classifier was also trained with the unscaled input data.

### 3.3. Dataset Creation

The acquisitions involved sixteen participants from 24 to 34. Non-expert participants were selected to obtain natural hand shape variability, avoid expert-level motor biases, and ensure that the recognition system generalizes effectively to real-world users who do not possess formal training in sign language. Participants followed a predefined motion sequence to ensure consistent acquisition and capture multiple viewpoints. This also reduced occlusions and simulated realistic variability. Although the sequence of individual gestures was the same, the order in which the gestures were performed was chosen randomly. The acquisition protocol was specifically designed to introduce motion exclusively to increase viewpoint and depth variability, rather than to encode temporal dependencies. Because the study focuses on the recognition of static gestures, the classification framework is intentionally frame-based. This ensures maximum compatibility with modular systems and maintains the low-latency operation required for real-time applications. By treating each frame as an independent observation, the system avoids the unnecessary computational overhead associated with temporal buffers, focusing instead on the discriminative features of the gesture itself.

The subjects began by rotating their hand along the axis of their arm, then waved their hand from right to left in a lateral movement and finally moved their hand toward and away from the camera. All the signs are executed in the upward torso region, where signs are generally expected to be recorded. The LIS sign space covers a relatively circumscribed area thus avoiding a dispersion in the execution of the sign that would make it difficult for the listener to perceive it [10]. The recordings were monitored in real-time to ensure the quality and completeness of the data. By following this protocol, the data collection aimed to generate a comprehensive and high-quality dataset of hand gestures to train a classifier that could be used in real-world situations. Indeed, the hand’s dynamic motion ensures the collection of various perspectives and angles of view to capture the changing depth information and space position.

To facilitate the participants’ familiarization with the specific configurations they would later encounter during the actual experiment, an augmented reality application for the Microsoft HoloLens 2 (Microsoft Corporation, Redmond, WA, USA) was developed. The application allowed even those without prior knowledge of sign language to engage with the material in an intuitive and interactive manner. The application was designed to guide users step-by-step through the gestures and sequences they were expected to learn, providing visual cues and spatial overlays that could be explored from multiple perspectives, thereby transforming the learning process into a fully immersive experience. Augmented reality allowed participants to interact with virtual elements while remaining aware of their environment. This bridged the gap between abstract instructions and tangible actions. By overlaying instructional content directly onto their surroundings, participants were able to contextualize the movements and signs within a spatial and situational framework that mirrored the conditions of the upcoming experiment, enhancing their cognitive engagement and fostering a deeper understanding of both the gestures themselves and the context in which they would be applied.

The augmented reality approach not only reduced initial anxiety and uncertainty but also encouraged participants to become more conscious of the relationship between the virtual instructions and the physical space they occupied, ultimately leading to a more prepared and confident performance during the subsequent experimental phase.

**Figure 5:**
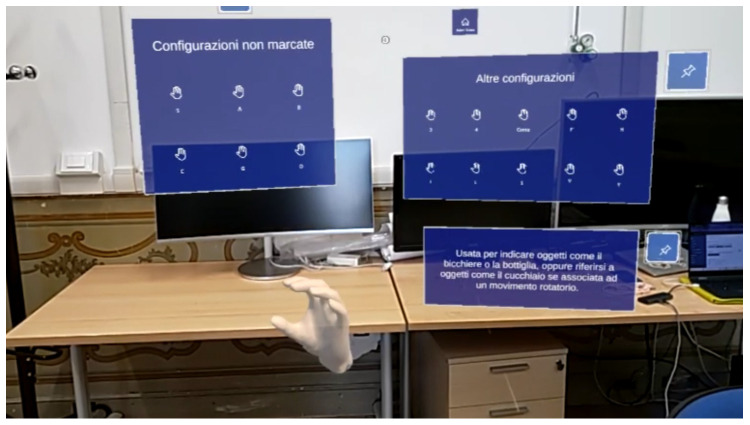
HoloLens application user interface to display different marked or unmarked configurations.

As illustrated in Figure 5, two different panels were displayed to the users, the six unmarked configurations on the left and ten further signs on the right.

**Figure 6:**
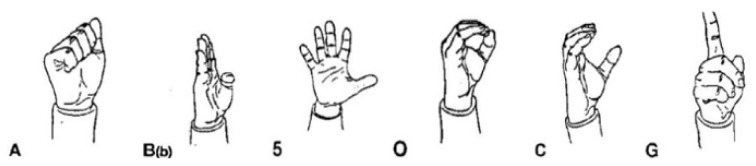
The six unmarked configurations.

Coherently, two different datasets were created. The first dataset consisted of 1200 frames capturing 200 frames for each of the six unmarked configurations (Figure 6):

- 5: Used to indicate large, open areas, suggest the transparency of surfaces, express the concept of plurality, or symbolize the number five.
- A: Used to indicate the idea of strength or power, hard objects, bodies that close in on themselves, or to symbolize a grip or grasp.
- B: Used to express possession, temporal (before, after) or spatial (in front of, behind) deixis, or for actions such as cutting.
- C: Used to indicate objects such as a glass or bottle, or to refer to objects such as a spoon when associated with a rotating movement.
- G: Used as a pronoun reference (I, you) or to indicate body parts or entities.
- O: Used to indicate circular elements such as rings, or when placed next to the mouth, to indicate gestures reminiscent of drinking.

**Figure 7:**
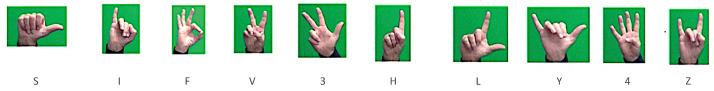
LIS configurations added to the initial unmarked configurations.

The second dataset comprised 3200 frames, adding ten further signs to the six unmarked configurations (Figure 7), always capturing 200 frames each.

- 3: Used as a free variation of configuration 5, it indicates the number three, flat or open surfaces, or three-pointed objects.
- 4: Used to indicate thin, parallel objects or to indicate the number four.
- F: Used to refer to round, thin objects, or to an empty set, or to say “okay”.
- H: Used to indicate the gripping of small, light objects or to represent flat, linear objects.
- I: Used as a variant of configuration B, it indicates small, thin objects or is used to introduce objects that begin with the letter J.
- L: Used to indicate the number two, to describe the concept of a pair, or to describe the limits of square surfaces.
- S: Used to represent the pouring of a liquid substance, indicate the number one, or for actions such as engraving, digging, or counting.
- V: Used to indicate objects with two protrusions, activities involving speaking, or to express verbs such as “to observe”.
- Y: Used to refer to all objects with two side protrusions, such as a telephone.
- Z: Used to express the concept of evil or otherwise convey a negative connotation.

### 3.4. Classification Algorithm

**Figure 8:**
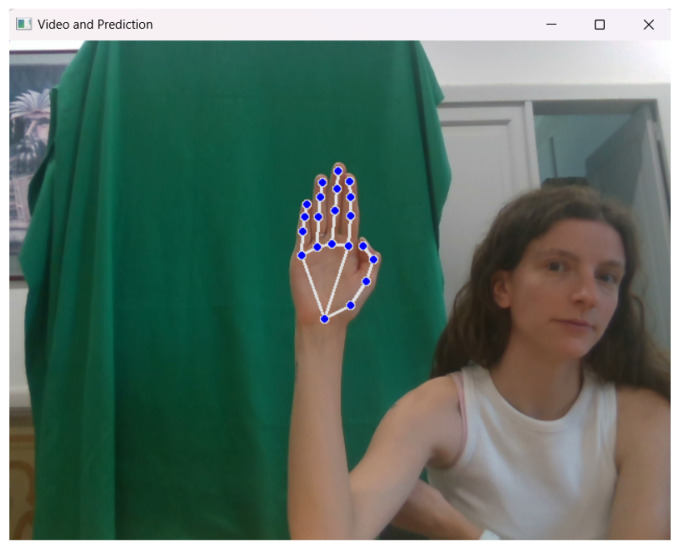
Graphic interface of the real-time system. Blue dots indicate the detected hand landmarks used for tracking.

A Support Vector Machine (SVM) classifier was employed for the gesture recognition task due to its robustness in high-dimensional feature spaces and its strong generalization capabilities when trained on relatively limited datasets [40], as is often the case for hand landmark-based gesture representations [41]. The dataset was partitioned using an 80–20 ratio into training and test sets. The dataset was split at the frame level, not at the subject level, with the analogous number of frames for each subject, i.e., 14 frames for each sign, with a 1-frame gap on some subjects to have exactly 200 frames per sign. Consequently, frames from the same participant may appear in both the training and test sets. This choice specifically evaluates configuration discriminability under intra-subject variability. It does not focus on signer-independent generalization. Frame-level splits prevail for static gesture datasets due to high visual consistency of configurations across users (e.g., MediaPipe landmarks normalize anatomy differences), unlike dynamic full-sign recognition where signer style induces stronger variability. Subject-independent splits are emphasized more for continuous signing (e.g., signer-based partitioning in Multi-VSL or low-resource SLR), but frame splits suffice for modular chereme tasks with non-expert signers [42]. During the data selection process (Figure 8), care was taken to maintain balanced class distributions among all the classes.

Prior to classification, all features were standardized to zero mean and unit variance in order to prevent scale-dependent bias and to ensure stable convergence of the SVM optimization process. A linear kernel was selected based on both empirical observations and application-oriented considerations. After standardization, the landmark-based feature vectors exhibited near-linear separability, for which linear decision boundaries provided stable convergence and high classification accuracy across both gesture sets. While non-linear kernels could potentially capture more complex class boundaries, they were intentionally not explored in this study in order to limit model complexity and to maintain a controlled experimental setting for analyzing the effect of depth information quality. Furthermore, linear SVMs offer lower computational cost and greater interpretability, making them more suitable for real-time scenarios, which represent the target application domain of the proposed framework. The regularization parameter was set to C = 1.0, providing a balanced compromise between maximizing the margin and minimizing classification errors, thus limiting overfitting while preserving discriminative power across gesture classes. Additionally, probability estimation was enabled to obtain posterior class probabilities, which are useful for confidence-aware decision making and potential integration with higher-level temporal or multimodal recognition frameworks. A fixed random state was used during training to ensure the reproducibility of the experimental results.

## 4. Results and Discussion

This section provides a comprehensive evaluation of the proposed SVM-based gesture recognition framework, highlighting the combined effects of depth information quality and gesture set complexity on classification performance.

**Figure 9:**
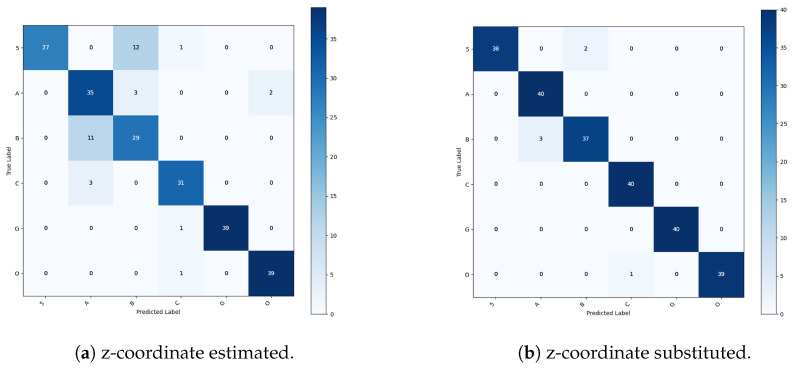
Confusion matrices referring to the datasets representing the six unmarked configurations.

In the recognition of the six unmarked configurations (Figure 9), the SVM trained using estimated depth coordinates achieves an overall accuracy of 83.8%, indicating that the framework is already effective under depth estimation constraints. The corresponding confusion matrix reveals structured misclassification patterns concentrated among geometrically similar hand configurations, most prominently between configurations 5 and B, as well as between C and O. These errors suggest that depth estimation inaccuracies affect geometric consistency. Thus, separability is reduced for configurations that differ primarily along the depth dimension rather than in planar shape. As a consequence, gestures characterized by subtle three-dimensional variations become partially overlapping in the learned representation. This interpretation is supported by the class-wise performance metrics, which show a marked disparity in recall across configurations. In particular, configuration 5 exhibits recall values in the range of approximately 67–70%, reflecting its frequent confusion with configuration B, whereas configurations such as G and O achieve perfect recall due to their more distinctive spatial structure and lower sensitivity to depth estimation errors. Precision follows a similar trend, with lower values for classes involved in mutual confusion, indicating that depth estimation errors affect both false negatives and false positives.

When real depth measurements acquired from an RGB-D camera are used for the same six-class task, classification performance improves substantially. Overall accuracy increases to 97.5%, corresponding to an absolute improvement of 13.7%, and the confusion matrix becomes almost entirely diagonal. At the class level, both recall and precision exceed 95% for all gestures, with most classes achieving 100% recall and precision, demonstrating a uniform and robust performance improvement. These results confirm that accurate depth information significantly enhances the discriminative power of the feature representation, enabling near-optimal classification even with a linear SVM.

**Figure 10:**
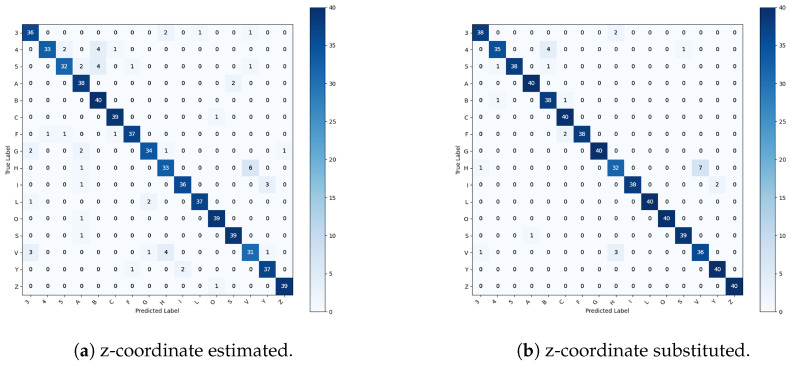
Confusion matrices referring to the datasets representing the sixteen configurations.

A similar but more pronounced behavior emerges in the more challenging scenario involving 16 hand configurations (Figure 10), where inter-class geometric differences are inherently more subtle and the classification task requires a larger number of decision boundaries within the same feature space. When estimated depth coordinates are employed, the SVM achieves an overall accuracy of 90.6%, indicating good baseline performance; however, the confusion matrix reveals a systematic increase in misclassifications among configurations that share similar planar hand shapes or differ predominantly along the depth dimension. Confusions between H and V stem from similar finger extension patterns. From certain viewpoints, depth estimation inaccuracies reduce the separation between parallel and slightly diverging finger configurations. Similarly, misclassifications between configurations 3 and G arise from their shared finger count and overall silhouette, with depth cues playing a critical role in distinguishing finger spacing and relative protrusion. The confusion observed between configurations 4 and B further highlights the limitations of estimated depth, as these configurations exhibit comparable palm orientation and finger alignment in two-dimensional projections, making accurate three-dimensional information essential for reliable discrimination. Collectively, these patterns indicate that as the number of configuration classes increases, depth estimation errors have a compounding effect on class overlap, particularly for gestures whose discriminative features are primarily encoded in fine-grained three-dimensional relationships rather than in gross hand shape. This increased ambiguity is reflected in the per-class recall distribution, which spans approximately 77–80% for gestures such as H and V to 100% for highly distinctive gestures such as B, Z, and O. Precision values mirror this behavior, with lower scores for gestures affected by inter-class overlap and higher values for geometrically unique configurations, indicating that depth estimation inaccuracies have a stronger impact as task complexity increases.

The use of real RGB-D depth measurements in the 16-class scenario leads to a further improvement in performance, with overall accuracy increasing to 95.6%, corresponding to an absolute gain of 5.0%. The confusion matrix shows a marked reduction in off-diagonal errors, and per-class recall consistently exceeds 90%, with the majority of classes achieving 95–100% recall. Precision values are similarly high, typically falling within the 95–100% range across all classes. Residual misclassifications are limited to gestures with strong intrinsic similarity. This suggests that remaining ambiguities arise from structural overlap within the gesture set rather than sensing noise or model limitations.

These results demonstrate that the proposed SVM-based framework is both robust and scalable, maintaining high accuracy when transitioning from a small to a larger gesture vocabulary.

To evaluate the robustness of the classification performance, descriptive metrics were supplemented with confidence interval (CI) estimations and two-sample proportion z-tests based on the respective test set sizes. These analyses aimed to determine whether the performance gap between estimated-depth and real-depth configurations was statistically significant. The results across both datasets indicate a consistent performance advantage for real-depth classification:

- Six-Configuration Dataset (N=1200): Estimated-depth classification achieved an accuracy of 83.8% (95% Wilson CI: 81.6–85.8%). In contrast, real-depth classification reached 97.5% (95% CI: 96.5–98.2%). A two-sample proportion z-test confirmed that this improvement is statistically significant (z=−11.51, $p=1.2\times 10^{-30}$).
- 16-Configuration Dataset (N=3200): Estimated depth yielded 90.6% accuracy (95% CI: 89.5–91.6%), while real depth achieved 95.6% (95% CI: 94.8–96.3%).

Despite the higher baseline for estimated depth in this larger set, the difference remained statistically significant (z=−7.89, $p=3.1\times 10^{-15}$). Table 2 summarizes the comparative metrics and statistical significance across both experimental setups.

While estimated depth coordinates enable acceptable recognition rates and represent a viable solution in low-cost or hardware-constrained scenarios, the consistent improvements observed with real RGB-D depth data confirm that depth quality plays a critical role in maximizing classification reliability. In particular, accurate depth information not only improves overall accuracy but also reduces inter-class performance imbalance, leading to more consistent precision and recall across all gestures. These findings highlight the importance of high-quality depth sensing in complex multi-class gesture recognition systems, while also confirming the effectiveness of the proposed approach under varying sensing conditions.

Despite the promising results obtained, this study presents some limitations that should be acknowledged when interpreting the findings. First, the proposed approach focuses exclusively on static hand configurations, without explicitly modeling temporal dynamics. Although the acquisition protocol introduced controlled hand movements to capture variability in viewpoint and depth, the classification itself operates on individual frames. Consequently, the system does not yet account for dynamic cheremes such as movement trajectories or transitions between configurations, limiting the adoption of the proposed approach to non-advanced LIS students. Second, data were collected from a relatively small number of non-expert participants within a restricted age range and under controlled environmental conditions demonstrating the feasibility of such an approach to recognize signs. However, a study for investigating the efficacy of the system in learning LIS is planned for a future work. Moreover, the current experimental design adopts a frame-level train–test split, which allows frames from the same participant to appear in both sets and therefore primarily assesses within-subject generalization. While this choice is suitable for analyzing configuration discriminability under intra-subject variability, it does not fully capture signer-independent performance, which is a critical requirement for real-world deployment, especially concerning dynamic signs. Future work will address this limitation by adopting subject-wise evaluation protocols, in which all samples from a given participant are excluded from the training set and used exclusively for testing, enabling a more rigorous assessment of generalization across different signers. Another limitation concerns the sensor dependency of depth information. The results showed that accurate RGB-D depth measurements significantly improve classification performance. However, this also implies that the robustness of the system is partially tied to the quality and reliability of the depth sensor or the estimation algorithm. RGB-D cameras are an effective choice for retrieving 3D information with low-cost devices but may suffer from noise, missing depth values, or performance degradation under challenging conditions such as strong ambient light or reflective surfaces, potentially affecting system reliability outside controlled environments.

## 5. Conclusions

This work presented a camera-based machine learning framework for the recognition of hand configurations in Italian Sign Language (LIS), addressing a key limitation of many existing sign language recognition systems that operate at the level of complete signs or isolated alphabet letters. By focusing on hand configurations as fundamental cheremic units, the proposed approach promotes a more modular and linguistically grounded representation of signed communication.

An SVM classifier was trained on hand landmarks extracted from RGB images using MediaPipe and enriched with depth information acquired from an RGB-D camera. Experimental results on two datasets comprising six unmarked and 16 standard LIS configurations demonstrate that the proposed method achieves high recognition accuracy and maintains robust performance as the gesture vocabulary increases. In particular, the comparison between estimated and sensor-acquired depth values highlights the critical role of accurate depth information in improving class separability, reducing inter-class confusion, and ensuring balanced precision and recall across all configurations.

The proposed system is designed to be compatible with real-time operation and relies on low-cost, widely available hardware, making it suitable for practical deployment in real-time applications. By achieving high accuracy in recognizing static hand configurations, this work establishes a critical and robust prerequisite for a full LIS translation system. Possible directions for further research will extend the present analysis by exploring more advanced classification strategies, including non-linear kernels and alternative learning paradigms, in combination with systematic hyperparameter optimization and cross-validation. In addition, further investigation will address signer-independent evaluation protocols and temporal modeling of dynamic gestures, with the goal of integrating configuration-level recognition into a more comprehensive machine vision pipeline for Italian Sign Language understanding and translation. As such, this research paves the way for transitioning from precise configuration-level recognition to the challenging task of continuous LIS translation.

## Figures and Tables

**Table 1:** Example of a data frame for the first landmark coordinates.

Frame Number	Label	Landmark 0 *x*	Landmark 0 *y*	Landmark 0 *z*
0	1	0.348731	0.313553	−3.706656 × 10^−7^
1	1	0.349573	0.312795	−3.710082 × 10^−7^

**Table 2:** Comparative accuracy and statistical significance across datasets.

Dataset Size (*N*)	Method	Accuracy	95% Wilson CI	*z*-Score	*p*-Value
1200	Estimated Depth	83.8%	81.6–85.8%	−11.51	$1.2\times 10^{-30}$
	Real Depth	97.5%	96.5–98.2%		
3200	Estimated Depth	90.6%	89.5–91.6%	−7.89	$3.1\times 10^{-15}$
	Real Depth	95.6%	94.8–96.3%		

## Data Availability

The raw data supporting the conclusions of this article will be made available by the authors on request.

## References

[1] La Grutta S., Piombo M.A., Spicuzza V., Riolo M., Fanara I., Trombini E., Andrei F., Epifanio M.S. (2023). The Relationship between Knowing Sign Language and Quality of Life among Italian People Who Are Deaf: A Cross-Sectional Study. Healthcare.

[2] Kuenburg A., Fellinger P., Fellinger J. (2016). Health Care Access Among Deaf People. J. Deaf Stud. Deaf Educ..

[3] James T.G., Varnes J.R., Sullivan M.K., Cheong J., Pearson T.A., Yurasek A.M., Miller M.D., McKee M.M. (2021). Conceptual Model of Emergency Department Utilization among Deaf and Hard-of-Hearing Patients: A Critical Review. Int. J. Environ. Res. Public Health.

[4] McKee M.M., Lin F.R., Zazove P. (2018). State of research and program development for adults with hearing loss. Disabil. Health J..

[5] Brenner J.M., Baker E.F., Iserson K.V., Kluesner N.H., Marshall K.D., Vearrier L. (2018). Use of Interpreter Services in the Emergency Department. Ann. Emerg. Med..

[6] Zahid H., Rashid M., Hussain S., Azim F., Syed S.A., Saad A. (2022). Recognition of Urdu sign language: A systematic review of the machine learning classification. PeerJ Comput. Sci..

[7] Koller O. (2020). Quantitative Survey of the State of the Art in Sign Language Recognition. arXiv.

[8] Ulrich L., Carmassi G., Garelli P., Lo Presti G., Ramondetti G., Marullo G., Innocente C., Vezzetti E. (2024). SIGNIFY: Leveraging machine learning and gesture recognition for sign language teaching through a serious game. Future Internet.

[9] Rasetto S., Marullo G., Adamo L., Bordin F., Pavesi F., Innocente C., Vezzetti E., Ulrich L. (2025). ReHAb Playground: A DL-Based Framework for Game-Based Hand Rehabilitation. Future Internet.

[10] Mazzaraco L. (2010). LIS e semiotica. Studi Glottodidattica.

[11] de Souza C.R., Pizzolato E.B. (2013). Sign Language Recognition with Support Vector Machines and Hidden Conditional Random Fields: Going from Fingerspelling to Natural Articulated Words. Machine Learning and Data Mining in Pattern Recognition.

[12] Katoch S., Singh V., Tiwary U.S. (2022). Indian Sign Language recognition system using SURF with SVM and CNN. Array.

[13] Podder K.K., Ezeddin M., Chowdhury M.E.H., Sumon M.S.I., Tahir A.M., Ayari M.A., Dutta P., Khandakar A., Mahbub Z.B., Kadir M.A. (2023). Signer-Independent Arabic Sign Language Recognition System Using Deep Learning Model. Sensors.

[14] Jintanachaiwat W., Jongsathitphaibul K., Pimsan N., Sojiphan M., Tayakee A., Junthep T., Siriborvornratanakul T. (2024). Using LSTM to translate Thai sign language to text in real time. Discov. Artif. Intell..

[15] Oudah M., Al-Naji A., Chahl J. (2020). Hand Gesture Recognition Based on Computer Vision: A Review of Techniques. J. Imaging.

[16] Ma X., Peng J. (2018). Kinect Sensor-Based Long-Distance Hand Gesture Recognition and Fingertip Detection with Depth Information. J. Sens..

[17] Rodríguez-Moreno I., Martínez-Otzeta J.M., Goienetxea I., Sierra B. (2022). Sign language recognition by means of common spatial patterns: An analysis. PLoS ONE.

[18] Bamwend J., Özerdem M.S. (2019). Recognition of static hand gesture with using ANN and SVM. Dicle Univ. J. Eng..

[19] de Smedt Q., Wannous H., Vandeborre J.P., Guerry J., Le Saux B., Filliat D. SHREC’17 Track: 3D Hand Gesture Recognition Using a Depth and Skeletal Dataset. Proceedings of the 3DOR-10th Eurographics Workshop on 3D Object Retrieval.

[20] Vo D.H., Huynh H.H., Doan P.M., Meunier J. (2017). Dynamic Gesture Classification for Vietnamese Sign Language Recognition. Int. J. Adv. Comput. Sci. Appl..

[21] Kakizaki M., Miah A.S.M., Hirooka K., Shin J. (2024). Dynamic Japanese Sign Language Recognition Through Hand Pose Estimation Using Effective Feature Extraction and Classification Approach. Sensors.

[22] Pansare J.R., Gawande S.H., Ingle M. (2012). Real-Time Static Hand Gesture Recognition for American Sign Language (ASL) in Complex Background. J. Signal Inf. Process..

[23] Nguyen Huu P., Phung Ngoc T. (2021). Hand Gesture Recognition Algorithm Using SVM and HOG Model for Control of Robotic System. J. Robot..

[24] Wachs J.P., Kölsch M., Stern H., Edan Y. (2011). Vision-Based Hand-Gesture Applications. Commun. ACM.

[25] Saini T., Kumari N. SignaSpectrum: AI-Driven Dynamic Sign Language Detection and Interpretation. Proceedings of the 11th International Conference on Reliability, Infocom Technologies and Optimization (ICRITO).

[26] Amangeldy N., Kudubayeva S., Kassymova A., Karipzhanova A., Razakhova B., Kuralov S. (2022). Sign Language Recognition Method Based on Palm Definition Model and Multiple Classification. Sensors.

[27] Zakariah M., Alotaibi Y.A., Koundal D., Guo Y., Elahi M.M. (2022). Sign Language Recognition for Arabic Alphabets Using Transfer Learning Technique. Comput. Intell. Neurosci..

[28] Kakoty N.M., Sharma M.D. (2018). Recognition of Sign Language Alphabets and Numbers based on Hand Kinematics using A Data Glove. Procedia Comput. Sci..

[29] Almeida S.G.M., Guimarães F.G., Ramírez J.A. (2014). Feature extraction in Brazilian Sign Language Recognition based on phonological structure and using RGB-D sensors. Expert Syst. Appl..

[30] Saravanan R., Veluchamy S. Sign Language Classification with MediaPipe Hand Landmarks. Proceedings of the International Conference on Energy, Materials and Communication Engineering (ICEMCE).

[31] Ameur S., Bouhlel M.S. A Comprehensive Leap Motion Database for Hand Gesture Recognition. Proceedings of the 7th International Conference on Sciences of Electronics, Technologies of Information and Telecommunications (SETIT).

[32] Tun M., Lwin T. (2019). Real-time Myanmar Sign Language Recognition System using PCA and SVM. Int. J. Trend Sci. Res. Dev. (IJTSRD).

[33] Xue Q., Li X., Wang D., Zhang W. (2019). Deep Forest-Based Monocular Visual Sign Language Recognition. Appl. Sci..

[34] Ariesta M.C., Wiryana F., Suharjito, Zahra A. Sentence Level Indonesian Sign Language Recognition Using 3D CNN and BiRNN. Proceedings of the 1st INAPR International Conference.

[35] Pietrandrea P. (2002). Iconicity and arbitrariness in Italian sign language. Sign Lang. Stud..

[36] Zabatani A., Surazhsky V., Sperling E., Moshe S.B., Menashe O., Silver D.H., Karni Z., Bronstein A.M., Bronstein M.M., Kimmel R. (2019). Intel® realsense^TM^ sr300 coded light depth camera. IEEE Trans. Pattern Anal. Mach. Intell..

[37] Lugaresi C., Tang J., Nash H., McClanahan C., Uboweja E., Hays M., Zhang F., Chang C.L., Yong M.G., Lee J. (2019). MediaPipe: A Framework for Building Perception Pipelines. arXiv.

[38] Lin Y., Jiao X., Zhao L. (2023). Detection of 3D Human Posture Based on Improved Mediapipe. J. Comput. Commun..

[39] Maculotti G., Ulrich L., Olivetti E.C., Genta G., Marcolin F., Vezzetti E., Galetto M. (2022). A methodology for task-specific metrological characterization of low-cost 3D camera for face analysis. Measurement.

[40] Wang P., Fan E., Wang P. (2021). Comparative analysis of image classification algorithms based on traditional machine learning and deep learning. Pattern Recognit. Lett..

[41] Xu H., Caramanis C., Mannor S. (2009). Robustness and regularization of support vector machines. J. Mach. Learn. Res..

[42] Dinh N.S., Nguyen T.D., Tran D.T., Pham N.D.H., Tran T.H., Tong N.A., Hoang Q.H., Le Nguyen P. (2025). Sign Language Recognition: A Large-scale Multi-view Dataset and Comprehensive Evaluation. 2025 IEEE/CVF Winter Conference on Applications of Computer Vision (WACV).

